# Measuring Efficiency and Productivity Growth of New Technology-Based Firms in Business Incubators: The Portuguese Case Study of Madan Parque

**DOI:** 10.1155/2015/936252

**Published:** 2015-03-22

**Authors:** A. Grilo, J. Santos

**Affiliations:** UNIDEMI, Departamento de Engenharia Mecânica e Industrial, Faculdade de Ciências e Tecnologia da Universidade Nova de Lisboa, Monte de Caparica, 2829-516 Caparica, Portugal

## Abstract

Business incubators can play a major role in helping to turn a business idea into a technology-based organization that is economically efficient. However, there is a shortage in the literature regarding the efficiency evaluation and productivity evolution of the new technology-based firms (NTBFs) in the incubation scope. This study develops a model based on the data envelopment analysis (DEA) methodology, which allows the incubated NTBFs to evaluate and improve the efficiency of their management. Moreover, the Malmquist index is used to examine productivity change. The index is decomposed into multiple components to give insights into the root sources of productivity change. The proposed model was applied in a case study with 13 NTBFs incubated. From that study, we conclude that inefficient firms invest excessively in research and development (R&D), and, on average, firms have a productivity growth in the period of study.

## 1. Introduction

The highly competitive environment of new technologies forces firms to seek mechanisms that enable them to achieve sustainable growth. Recent years have seen the emergence of business incubators all over the world. Incubators can play a key role in supporting new technology-based firms (NTBFs) by betting on innovation as a way to help the creation and development of these firms. However, it is still unclear whether the mission of the incubators to encourage the growth of NTBFs has been successful [1]. Since NTBFs are the general typology of incubated firms, it is important to ensure efficient management of the available resources. Costs arising from the lack of efficiency can compromise the survival and growth of incubated firms, regardless of their business area. This situation forces firms to minimize their costs while continuing to provide quality and diversity products. Therefore, performance evaluation and benchmarking could help the NTBFs to become more productive and efficient by avoiding their untimely death. Although decisions about NTBF's strategy are ultimately from the entrepreneurs' responsibility, business incubators may provide efficiency and productivity benchmarking tools in their guidance role for incubated firms.

Data envelopment analysis (DEA) has been highlighted as an assessment tool of technical efficiency in organizations. The DEA allows evaluating the relative efficiency of a combination of units that convert multiple inputs into multiple outputs. For a given set of input and output variables, DEA produces a single comprehensive measure of performance (efficiency score) for each unit [2]. To measure the productivity change over time, this paper suggests a DEA-based Malmquist productivity index methodology [3]. Some studies focus on the importance of efficiency evaluation in the technology sector through the use of DEA, namely, with respect to R&D activities (see [4–8]). However, there is a shortage of research into the subject in the context of incubated firms.

The aim of this paper is to create a model for estimating the technical efficiency and productivity growth of NTBFs located in a business incubator during the period 2009–2011. The proposed model aims to help decision-making about an NTBF's strategy, and though ultimately this is mainly entrepreneurs' responsibility, business incubators may provide efficiency and productivity benchmarking tools in their guidance role for incubated firms. Managers of NTBFs and business incubators' managers may adopt the proposed model as an internal benchmark management procedure in order to evaluate the relative position of each firm to the efficient frontier, thus enabling a relative orientation in terms of their productivity. Based on these comparisons, both across companies and over a period of time, it will be possible for firms with more specific sources on how to address improvements in terms of management in a macroperspective.

The paper is organized as follows.  Section 2 presents the state of art regarding the main characteristics for business incubators and some of their challenges regarding the evaluation of performance.  Section 3 describes the concepts and models related to DEA, along with the Malmquist index. The proposed model to evaluate efficiency and productivity growth of NTBFs is presented in Section 4 along with the description, analysis and discussion of the results of the case study of Madan Parque, an incubator in Portugal. In Section 5 conclusions are presented.

## 2. Incubating New Technology-Based Firms (NTBFs)

The main characteristics of NTBF are [9–11] (a) high percentage of engineers and researchers among employees; (b) fast growth rate and a global market for their products; (c) innovation and advanced technology in the products and services; (d) investment of at least 3% of their revenue in research and development (R&D) activities.

National and regional governments have launched several political programs and policies over the years seeking to develop a nurturing environment for NTBFs. These political measures are often a way to revitalize European regions that have been in economic decline and where there is a belief that NTBF can reverse that downwards trend [12]. Science & Technology Parks (STP) and especially business incubators within STP have had a major role in providing infrastructures for the execution of these national and regional economic policies, with public and private funding. Such efforts have increased considerably due to the recognition of STPs' importance in the development and maturity of NTBFs [13].

Despite NTBFs performance and contribution to the economy, there are factors that may jeopardize their economic potential, for example, the management capacity and the sales ability of their marketing drive, as entrepreneurs often have mainly technological skills and competences. Thus, NTBFs' success often depends on the quality of the management resources made available by business incubators in STP and the sources of capital [14]. NTBF entrepreneurs are more likely to start and grow their companies in SCT business incubators than outside them [15]. Siegel et al. [16] analyzed research productivity in companies located in STP business incubators compared with similar companies located outside STP and their findings demonstrate that NTBFs located in STP are more effective in terms of creation of new products, services, and patents.

According to Monck et al. [14], NTBF performance indicators can be divided into two groups:* inputs* measurement for high-technology activity, such as the number of qualified employees and the R&D effort characterized by investment in R&D as a percentage of overall sales and* output* measurement, such as growth rate and the number of patents and technological innovations. Walter et al. [17] examine academic spin-offs in STP business incubators and find that sales growth rate is a fundamental indicator for measuring the performance of this type of company as it can measure NTBFs management autonomy to explore the market and thereby validate the acceptance of the customers to the R&D results, as does also advocate Oakey [18]. To measure the internal efficiency, the authors propose the ratio of sales per employee.

Wang et al. [7] developed a model to measure the performance of NTBFs through four perspectives: financial, customer, internal processes, and growth perspective. The authors used a combined approach of hierarchical balanced scorecard (HBSC) and nonadditive fuzzy. The aim was therefore to develop a tool for improving performance measures through HBSC in complex environments and high competitiveness. The HBSC serves as a bridge between financial and nonfinancial perspectives and is an integrated system of performance measurement, combining the objectives of the organizations and other traditional functional areas of corporate strategy. For this, the authors use two key performance indicators (results-oriented and company development) in order to measure the strategy's implementation. Their study demonstrates the limitations that may exist in an HBSC survey of performance measures and thus contributes to the improvement of the effectiveness and efficiency of management.

## 3. Measuring Efficiency and Productivity Growth 

### 3.1. Data Envelopment Analysis

Data envelopment analysis (DEA) measures the relative efficiency of decision-making units (DMUs) with multiple inputs and multiple outputs using a linear programming based model. The technique is nonparametric because it requires no assumption about the weights of the underlying production function [19]. Furthermore, DEA does not require prescribing the functional forms of the relationships between inputs and outputs that are needed in statistical regression approaches, and the variables can be measured in different units [20].

The set of efficient DMUs that form the efficient frontier can be identified. Thus, DEA is also a powerful benchmarking technique since it allows measuring the level of efficiency of nonfrontier units and identifying benchmarks against which such inefficient units can be compared [21].

In the literature, two DEA models are commonly used. The initial basic frontier model, known as the Charnes, Cooper, and Rhodes (CCR) model [22], is built on the assumption of constant returns to the scale (CRS) of activities. The second model, known as Banker, Charnes, and Cooper (BCC) model [23], is built on the assumption of variable returns to the scale (VRS) of activities. There are two versions for both the CCR and the BCC models to determine the efficient frontier. One is input-oriented and the other output-oriented [24].

The output-oriented score (*ϕ*) will be greater than or equal to 1, and that *ϕ* − 1 is the proportional increase in outputs that could be achieved by the unit under evaluation, with input quantities held constant. It is noted that 1/*ϕ* defines a technical efficiency score that varies between 0 and 1 [25]. The existence of input and output slacks shows that additional input reduction or output production is needed in order to make the DMU efficient [2].

According to Banker and Thrall [26], the BCC model allows one to decompose the* technical efficiency* (TE), obtained through the CCR model in* scale efficiency* (SE) and* pure technical efficiency* (PTE). The* score* CCR is denominated TE, and the* score* obtained through the BCC model measures PTE. For a specific DMU, if the technical efficiency* scores* differ in the CCR model and the BCC model, then the DMU presents an inefficiency of scale. Scale efficiency evaluates the capacity of a unit being produced in CCR. If the* scores* of the two models are equal, then the DMU is operating under CCR, that is, in the most efficient scale of production. This scale inefficiency may be computed through the score difference of the technical efficiency of BCC and CCR. A DMU BCC efficiency is always greater than or equal to the efficiency measured in the CCR model [25].

The SE is defined by the ratio of TE to PTE:

(1) $$\begin{array}{c} \text{SE}=\frac{\text{TE}}{\text{PTE}}=\frac{\phi_{\text{CCR}}^{*}}{\phi_{\text{BCC}}^{*}}. \end{array}$$

SE is always lower than 1. Expression (1) is equivalent to TE = SE × PTE. This decomposition describes the sources of inefficiency, which may be caused by an inefficient operation from the DMU (PTE), by disadvantageous conditions under which the DMU is operating (SE), or both [20].

DEA has also been widely applied to different industries, and a number of different DEA models have been developed and improved based on the original DEA model (see [2, 21, 24, 27–31]).

### 3.2. Malmquist Productivity Index

The Malmquist productivity index (MI) [32], based on DEA models, is one of the prominent indices for measuring the relative productivity change of DMUs over time. From the combination of the inputs and outputs of a DMU in periods *t* and *t* + 1, it is possible to determine whether the variation in the performance of this DMU is due to technical efficiency change (TEC) of each DMU and/or technological change (TC).

Compared to other indices, the Malmquist productivity index presents some important characteristics and properties. The Malmquist productivity index can be useful in situations in which the objectives of managers differ, are unknown, or are difficult to implement, since it does not require any assumption regarding the cost minimization or profit maximization [33]. Moreover, an assumption associated with application of MI is the existence of a competitive market, which encourages businesses to implement effective strategies [25]. The calculation of the MI requires measurements of two different time periods and two grouped periods. The measures of the two different time periods can be obtained through the DEA CCR model.

Färe et al. [34] specify an output-based MI calculated for year *t* and *t* + 1 technologies defined by (2). Consider

(2) $$\begin{array}{c} M_{0}={\left[\frac{D_{0}^{t}\left(x_{0}^{t+1},y_{0}^{t+1}\right)}{D_{0}^{t}\left(x_{0}^{t},y_{0}^{t}\right)}\cdot \frac{D_{0}^{t+1}\left(x_{0}^{t+1},y_{0}^{t+1}\right)}{D_{0}^{t+1}\left(x_{0}^{t},y_{0}^{t}\right)}\right]}^{1/2}. \end{array}$$

The above measure is actually the geometric mean of two Malmquist productivity indices [1]. When *M*_0_ > 1, it indicates productivity gain; when *M*_0_ < 1, it signifies productivity loss; and *M*_0_ = 1 means no change in productivity from *t* and *t* + 1 [5]. The MI can be decomposed into two components: the first component is the technical efficiency change (TEC); and the second component is the shift in the frontier or the technological change (TC) between periods *t* and *t* + 1 [3]. Consider

(3) M0=D0t+1x0t+1,y0t+1D0tx0t,y0t ×D0tx0t,y0tD0t+1x0t,y0t·D0tx0t+1,y0t+1D0t+1x0t+1,y0t+11/2, M0=TEC×TC.

The ratio outside the brackets measures the change in relative efficiency; that is, it is also a measure of how close the DMU is to the frontier in period *t* + 1 compared with period *t*. If TEC = 1, the DMU has the same distance in periods *t* + 1 and *t* from the respective efficient frontiers. If TEC > 1, the DMU has moved closer to the period *t* + 1 frontier than it was to the period *t* frontier, and if TEC < 1, the converse occurs. The bracketed term is the index of change in technology between two periods. If TC = 1, it indicates no shift in technology frontier; a value of TC < 1 indicates technological regress; TC > 1 indicates technological progress and is considered to be an evidence of innovation [19, 34].

In relation to the returns to scale assumption, the MI must be calculated in a first step on the basis of CRS, since, if measured according to VRS, the measurement obtained is inaccurate [35]. The TEC and TC indices are obtained under the assumption that the DMU operates according to CRS, that is, assuming that DMU is operating in an optimal scale. So, to deal with more realistic cases with VRS, the TEC calculated under the assumption of CRS technology can be further decomposed into pure technical efficiency change (PTEC) and scale efficiency change (SEC) [36]. SEC quantifies the productivity gain or loss associated with a production unit, evaluating whether movements inside the frontier are in the right direction to attain the CRS point, where changes in outputs result in proportional changes in inputs [37]. While the TEC term is associated with efficiency change calculated under CRS, the PTEC is the efficiency change calculated under VRS. In this case, MI would comprise three components [34]:

(4) $$\begin{array}{c} M_{0}=\text{PTEC}\times \text{SEC}\times \text{TC}. \end{array}$$

According to Grosskopf [36], a PTEC is defined as

(5) $$\begin{array}{c} \text{PTEC}=\frac{D_{0\text{VRS}}^{t+1}\left(x_{0}^{t+1},y_{0}^{t+1}\right)}{D_{0\text{VRS}}^{t}\left(x_{0}^{t},y_{0}^{t}\right)}. \end{array}$$

SEC presents the following formulation:

(6) $$\begin{array}{c} \text{SEC}=\frac{D_{0\text{CRS}}^{t+1}\left(x_{0}^{t+1},y_{0}^{t+1}\right)/D_{0\text{VRS}}^{t+1}\left(x_{0}^{t+1},y_{0}^{t+1}\right)}{D_{0\text{CRS}}^{t}\left(x_{0}^{t},y_{0}^{t}\right)/D_{0\text{VRS}}^{t}\left(x_{0}^{t},y_{0}^{t}\right)}. \end{array}$$

While the TEC refers to the changes in technical efficiency calculated under CRS, the PTEC corresponds to changes in technical efficiency with regard to VRS and represents the changes resulting from efficiency improvements in operations and management activities. This decomposition allows us to contemplate situations in which a DMU may be technically effective, since the volume of production uses the least amount of resources, but not operating at the optimal scale production. SEC shows the movements inside the boundary that are in the right direction to reach the CRS point at which the output changes result in proportional changes in inputs [36].

### 3.3. NTBF and DEA

In recent years, the application of DEA as an assessment tool for NTBFs has grown, especially with regard to the selection of projects for R&D, evaluation of efficiency of R&D processes, or factors affecting the results of R&D. It appears, therefore, that greater emphasis has been given to evaluation activities within the NTBF on the whole, leaving aside the assessment of the efficiency of NTBF.

Lu et al. [6] applied the DEA to study the performance of high-tech industries in R&D. According to these authors, the main factor of success in high-tech companies is to increase the efficiency and performance in R&D. The inputs used in their study were company assets, expenditure on R&D, number of employees, and the number of researchers directly linked to R&D. With regard to outputs, the authors considered number of patents, export volume, return on investment, and sales volume. The results obtained in their study help managers make decisions that make R&D more efficient and innovative.

Chen et al. [8] studied the application of DEA in performance evaluation in R&D companies in the field of computers and peripherals located in STP business incubators. In their study, the CCR and BCC models with three inputs and two outputs were used. The inputs considered were age of the firm, capital expenditure on R&D, and number of employees. With regard to the outputs, annual sales, and the number of patents were used. The authors concluded that the performances differed significantly between the various companies, although the vast majority of firms are technically efficient. Chen et al. [38] assessed the performance of six high-tech industries located in an STP. These authors used four inputs: number of employees, working capital, investment in R&D, and the area occupied. Two outputs were considered: annual sales and number of patents. In addition to studying the efficiency of the six industries each year individually through the CCR and BCC models, the authors used the MI to examine their growth over time.

Studies of performance in R&D NTBFs mostly use the same inputs and outputs, varying depending on data availability, and allow us to highlight the importance of R&D in NTBFs. Despite its drawbacks, the number of patents filed by a company continues to be widely used as a way to measure the level of technology diffusion. However, for many companies, introducing new products and services in the market is the most appropriate output of R&D [39]. For example, Chakrabarti [40] uses the number of patents not only as a measure of output of R&D, but also as the launch of new products and services by businesses. The author states that, for the growth of companies in some industries associated with designing new products, patents have no effect on sales growth.

DEA can help managers to identify NTBF sources of inefficiency, and the best ways to improve performance based on best practices of reference units. DEA does not provide specific information on corrective actions needed to improve business performance, but focuses, rather, on analyzing the reasons why a DMU is inefficient. Thus, managers have the task of evaluating the feasibility of the practical application of the proposed targets for the inputs and outputs [41]. The literature review carried out as part of this study showed that despite the many studies applying DEA to evaluate the efficiency of R&D, the DEA technique to evaluate the efficiency of NTBF in incubators at a more macroscale is not reported.

## 4. The Case Study of Madan Parque

### 4.1. Characterization of Madan Parque

To explore the applicability of the DEA and the MI in business incubators in STP, a case study was designed. To collect data, questionnaires were applied to firms incubated in Madan Parque, a business incubator located in Almada, Portugal. Madan Parque was founded in December, 1995, with the Faculty of Science and Technology of the New University of Lisbon (FCT-UNL) as the primary equity partner. The main service of Madan Parque is business incubation. The Parque provides modular office spaces equipped with telephone, electricity, air conditioning, and internet access, as well as access to common spaces, services, and joint activities. By 2012, there were 55 incubated companies that generated 195 jobs. Regarding all companies incubated in Madan Parque, the aggregate turnover was € 6,550,000, with a total investment in R&D of € 450,000, 25 brands registered, 8 national patents, and 3 international patents.

As it was intended to analyze the data available not only from the most recent year, but also from the two previous years, it was decided to restrict the analysis to firms operating in Madan Parque between the years 2009 and 2011, ignoring the companies that started or ended activity in this period. This condition restricted the sample to 21 companies. It is important that the DMUs included in the analysis are homogeneous. Thus, the DMUs considered in this work are essentially start-ups/spin-offs incubated from a similar technological baseline. Discarding firms with incomplete data resulted in a final sample of 13 firms.

Tables 1, 2, and 3 report the data collected for the years 2009, 2010, and 2011, respectively.

The data for the year 2011 (Table 3) were analyzed in order to obtain the coefficients of correlation between variables, thus eliminating redundant information. We chose to analyze only the data from the year 2011 because it was the most recent year of the sample.  Table 4 shows the matrix of correlations between inputs and outputs.

If two possible inputs present a high correlation, this may indicate that the inclusion of both is useless. The analysis of Table 4 shows that space costs are strongly correlated with the total number of employees, so the space costs variable was excluded from the analysis. The space costs are an indicator of firm size with respect to the occupied office area, so it is natural that the higher the amount of space costs, the greater the number of employees. Similarly, the salary costs have a strong correlation with the number of employees. In this case, we chose to leave out the total number of employees variable, since the pair input/output that has a higher correlation coefficient is the pair salary costs/sales. The correlation coefficients for the outputs do not show high values among themselves. To check if indeed there is any output variable that does not contribute significantly to the efficiency score, scenarios were developed with the two already selected inputs (salary costs and investment in R&D) and three output variables.  Table 5 summarizes the sensitivity analysis performed by different combinations of inputs and outputs. We used the* Data Envelopment Analysis Online Software* (DEAOS) and data of Table 5 for the average efficiency scores of the 13 DMUs in each scenario, applying the BCC output-oriented model. Thus, we intend to evaluate which variables contribute the most to the efficiency and which do not add value to the analysis, adding in the end the fewest possible variables.

We concluded that the output that contributes the least to the average efficiency of DMUs is the number of “clients,” so this variable is excluded from the analysis, leaving the model with four variables, two inputs, and two outputs.

**Figure 1:**
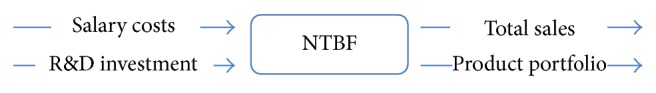
Final* inputs* and* outputs* of the DEA model.

After performing the sensitivity analysis, the variables that were considered the most appropriate for the DEA model, as shown in Figure 1, were selected.

The selected variables preferably take low values of input and high output values.

### 4.2. Analyzing and Discussing Results of DMUs Efficiency Using the DEA-BCC Model

The DEA-BCC model was applied again using the online software DEAOS to obtain the efficiency scores of 2009, 2010, and 2011, which were analyzed separately, as is shown in Table 6. On average, the 13 DMUs have an efficiency increase from year to year, which indicates a better mix of inputs consumed and outputs produced over the three years analyzed. DMUs with efficiency scores higher than 1 are considered inefficient and that the higher the value, the more inefficient the DMU. DMUs 1, 2, 4, 5, and 12 are efficient over the three years since they have an efficiency score equal to 1. DMU 3 shows a large increase in efficiency score from 2009 to 2010 but falls to a lower score in 2011. DMUs 6 and 8 have the same pattern as DMU 3. DMU 7 shows a considerable increase in the efficiency score in 2010 and achieves optimal efficiency in 2011. DMUs 9 and 10, by contrast, have a slight reduction in the efficiency score in 2010 and 2011 compared to that for 2009. DMUs 3, 6, 8, 9, 10, and 11 have efficiency scores higher than 1, and thus are considered ineffective according to the output-oriented model. These DMUs can reduce inefficiency through the proportional increase in their outputs. DMUs 1, 2, 4, 5, 7, 12, and 13 are considered efficient, since they have efficiency scores equal to 1, and the respective slacks are zero. These are therefore the best performing DMUs in the group.

Efficiency scores and the slacks shown in Table 7 allow us to identify the sources of inefficiency of DMUs already identified as inefficient for the year 2011. It is thus possible to identify specific situations of excessive use of resources and/or lack of results. Data illustrate the absence of DMUs with low efficiency, that is, with an efficiency score equal to 1 and nonzero slacks. The inefficient DMUs, that is, those with efficiency score greater than 1, reveal a shortage of results in all outputs. In this case, the sources of inefficiency differ whether DMUs have slacks or not. Inefficient DMUs that have slacks with zero values, in both the inputs and outputs, indicate that the lack of results for each output is contributing equally to inefficiency. Hence, for these DMUs to become efficient, a proportional increase of all outputs should occur, linked to the efficiency score. DMU 10 is the only one that in this situation should increase its outputs by 22% to become efficient. However, if there are nonzero slacks in inefficient DMU outputs, a proportional increase of outputs is insufficient to make the DMU efficient, since there is a lack of results from one (or more) output(s) contributing in particular further to the inefficiency. Thus, in addition to the proportional increase of all outputs, a further increase in outputs with nonzero slack is necessary. It is also possible that there are nonzero input slacks, suggesting that resources are being used in excess and that the DMU should therefore also make a corresponding reduction in the amount of slack in the input to move the DMU to the efficient frontier. DMUs 3, 6, 8, and 9 have considerable slacks in the variable “R&D Investment,” while DMU 11 has considerable slack in the variable “salary costs.” For example, DMUs 3 and 9 must proportionally increase their outputs, particularly, the “product portfolio,” as it has nonzero slack. Simultaneously, these DMUs should reduce their investment in R&D. This might seem contradictory, but it reinforces the idea that DMUs 3 and 9 are using too many of their resources for the results obtained. Thus, investment in R&D applied by DMUs 3 and 9 is not reflected in the results obtained in the same way as with the other DMUs. In this situation, managers should investigate the reasons for this excessive spending on R&D and improve its processes, producing more with fewer resources.

Analyzing the sources of inefficiency discussed above, it is possible to quantify the degree to which a DMU should increase its outputs or decrease its inputs to become efficient. In general, it is possible to set targets for the input and output variables of each DMU, which are in Table 8.

Note that these targets serve only as a diagnosis and that it is the responsibility of corporate managers to set realistic strategies to address the sources of inefficiency.

The classification of some DMUs as efficient or inefficient can be perceived immediately when examining the data. However, the classification of DMUs as efficient is not always intuitive and depends on the combination of its inputs and outputs and their comparison with the other DMUs. Units that could be considered efficient at the outset might not be efficient because there are units with a similar combination of inputs and outputs but can perform better. An example of this is the case of DMU 11, which has quite satisfactory results in both sales volume and product portfolio level, but it is classified as inefficient because it is the unit that has the most salary costs. Analyzing the value of the efficiency score for this unit, we can calculate that besides a 5% increase in its outputs, it would also be necessary to reduce salary costs by approximately € 109,263.

Moreover, the DEA provides information on the sources that cause such inefficiencies, which can be very useful for managers in identifying the factors that are deviating from the DMU's optimal performance. These work output-oriented inefficiencies are related not only to impaired production values, but also to the overuse of a particular input or poor production of an output. In all of the inefficient DMUs, we see the existence of at least one input or output that contributed especially to the inefficiency.

It is possible that, in the particular case under study, some of the observed quantitative targets are unreasonable for companies due to the fact that they do not already possess a high level of maturity, which may limit them in developing new products. Although the goals outlined may be of interest for managers to have a better perception of excessive costs and productive needs, they must not focus on those values only.

Finally, DEA identifies the benchmarks that inefficient units should take as an example to achieve their goals and become efficient. Inefficient units should identify the reasons why they cannot operate efficiently and realize what competing units do best and adapt those practices to their own unit.

### 4.3. Analyzing and Discussing of DMUs Efficiency-Malmquist Productivity Index

Initially, we applied the BCC model, in which the efficiency scores for each DMU in the years 2009, 2010, and 2011 were analyzed independently. The results allowed us to identify for each DMU improvement or a step backward in efficiency over the three years. However, it was not possible to identify the factors underpinning the improvement of their performance. The application of MI to the same data set yielded not only the proportion of the productivity gains of each DMU from year to year, but also the identity of the components that were the source of those gains. One of the components analyzed under MI was PTEC, which is calculated in considering VRS. Thus, changes in technical efficiency identified in the MI were in accordance with the assumption of VRS, that is, PTEC, and should be consistent with the changes that occurred in the BCC model.

For example, DMU 6 increases its efficiency score of 1,25 (year 2009) to 1,12 (2010); that is, the increase is 11.25%. Examining the PTEC of the same DMU concerning MI in 2009-2010, we see that there is a change in pure technical efficiency of 11.6%, which is quite close to the value obtained from the BCC model. This consistency prevails in the remaining DMUs. According to Barros and Alves [42], improvements in pure technical efficiency indicate that there was an investment in the company organizational factors, which may include a better balance between inputs and outputs, investments for marketing or quality improvements.

The BCC model indicated that DMUs 1, 2, 4, 5, and 12 are efficient over the three years, since they have an efficiency score that is equal to 1. When analyzing the data component of PTEC in 2009-2010 and 2010-2011, it is confirmed that there are no changes in pure technical efficiency for DMUs mentioned, since PTEC is equal to 1. This comparison confirms that when the aim is to evaluate the performance of a group of DMUs over time, the interpretation of the components resulting from the decomposition of the MI is more intuitive. Moreover, MI has the advantage of identifying the sources that contribute most to the productivity gains by calculating various indices.

The evolution of the performance of DMUs between the year 2009 (period *t*) and 2010 (period *t* + 1) shows an increase of 34.7% in the mean productivity of the 13 DMUs. The main contribution to this result is the increase of 24.8% in TC greater than the 10.3% increase for the TEC. The innovation was therefore the main source for the recorded mean productivity gains, suggesting that the adoption of new technologies by the DMUs led to considerable improvements. The TEC is the result of multiplying PTEC and SEC. On average, improvements in PTEC, that is, operations and management activities, are the main reason for the improvements in TEC. The average value of PTEC, which measures changes in technical efficiency under VRS, indicates that there was an improvement of 16.9% over the period.

From the five components analyzed in Table 9, only the SEC component has an average value below 1, which suggests a worsening of the scale efficiency. This situation indicates that the DMUs are operating above or below the optimal range, that is, they are having a too high cost for what they produce, or, conversely, they could increase production and reduce costs. Regarding the TEC, we see that DMUs 1, 6, 8, 9, 10, and 13 decreased their technical efficiency between 2009 and 2010. This suggests that these DMUs in 2010 are further away from the efficiency frontier in that period than they were in 2009. Moreover, DMUs 2, 3, 5, and 7 showed an increase in their technical efficiency, with special emphasis on DMU 3. Thus, these DMUs are closer to the frontier in 2010 when compared to the frontier of 2009. DMUs 4, 11, and 12 show the values of TEC that are equal to 1, so that there were no changes in technical efficiency from 2009 to 2010. Analyzing TC data, only DMU 11 has a value lower than 1, that is, technological regression. All other DMUs have TC values greater than 1, and therefore, between 2009 and 2010, there was a positive change in their technological frontier; that is, there was technological progress. This means that, for a given level of input, it is possible to obtain a higher level of output in 2010 than in 2009. This is due to the expansion of the frontier between the two periods.

Relative to MI, data suggest that DMUs 11 and 13 decreased their productivity, since they have values lower than 1 for this index. The remaining DMUs increased their productivity between 2009 and 2010 (MI > 1). It is interesting that the DMUs that show decreases of technical efficiency, with the exception of DMU 13, managed to overcome this situation with very positive changes in their technological frontiers, which contributed strongly to the productivity gains recorded. Regarding PTEC, DMUs 2, 3, 6, 7, and 8 present management improvements that translate into increased productivity. With the exception of DMUs 9, 10, and 13, all the others improved or maintained their PTEC between 2009 and 2010. With respect to SEC, we find that DMUs 2, 3, 5, and 7 increased their scale (size) in this period since they have values higher than 1. DMUs 4, 11, and 12 do not have scale issues and are operating on the frontier of CRS (optimal scale).

The analysis of the period 2010-2011 (Table 10) shows that the average productivity gain of the 13 DMUs was 65.5%, almost double that of the 2009-2010 period. Again, technological progress contributes considerably to productivity improvement, with an increase of 39.5%. This highlights the focus of DMUs in innovation with the introduction of new technologies in their processes. Once again, the SEC is the only component to register a negative average change between 2010 and 2011. However, its value is close to 1, so that, on average, the 13 DMUs are operating very close to their optimum level.

At the individual level, we should highlight DMUs 1 and 7, which have very high productivity gains. In the case of DMU 1, TC and TEC components contribute almost equally to the gains, while the DMU 7 improvements in technical efficiency played an important role in determining its achievements. DMU 3, which had considerably increased its productivity between 2009 and 2010 and dropped its productivity in 2011 due to the deterioration of technical efficiency. This decrease is probably due to the substantial reduction of its turnover between 2010 and 2011.

Since, for all DMUs except DMU 7, TC > TEC, we confirm that the productivity improvements are strongly due to technological progress. PTEC values show that the relationship between inputs and outputs in DMUs 3, 6, 8, 9, 10, and 11 worsened between 2010 and 2011; that is, these DMUs in 2011 were farther away from the VRS frontier formed by the reference DMU compared to the frontiers of 2010.

The number of DMUs that have productivity gains in 2010-2011 is lower than that in the 2009-2010 period. However, as mentioned above, the average improvements were approximately doubled. As such, it is concluded that although there are fewer DMUs with productivity gains, the improvements for these DMUs are quite positive (DMU 1 and DMU 7). In 2011, there are DMUs that suffered a considerable decrease in productivity compared to 2010, but there are also DMUs with a considerable growth, though these are fewer. Among the DMUs, with a fall in productivity in 2011, are DMUs 3, 8, and 9, which have their sales decline compared to the previous year and, in addition, there is an increase in their costs. The economic situation of Portugal at that time may help to explain this situation, with companies experiencing major difficulties in selling their products and seeking to counter this problem with greater investment in innovation.

The application of MI to collected data allows us to explore changes in productivity. With regard to the values obtained by the MI, we found that during both the period 2009-2010 and 2010-2011, gains in productivity were primarily due to changes in technology. These changes can be interpreted as investments in new technologies, which may include new methodologies, procedures, or techniques in order to improve results. The technological expansion could also mean that companies have improved their productivity by technical experience of its employees, taking advantage of modern facilities and equipment.

## 5. Conclusion

Business incubators can play a major role in helping to turn a business idea into a technology-based organization that is economically efficient. Performance evaluation and benchmarking could help the NTBFs to become more productive and efficient by avoiding their untimely death. However, there is a shortage in the literature regarding the efficiency evaluation and productivity evolution of the new technology-based firms (NTBFs) in the incubation context. Although decision-making about NTBF's strategy is ultimately the entrepreneurs' responsibility, business incubators may provide efficiency and productivity benchmarking tools in their guidance role for incubated firms. Hence, it is recommended for managers of NTBFs and business incubators' managers to adopt an internal benchmark management procedure in order to evaluate the relative position of each firm to the efficient frontier.

To explore the ability of DEA models to help NTBF in business incubators, a case study of Madan Parque, in Lisbon, Portugal, was conducted. Of the 13 units studied, six were identified as inefficient and probably these results were influenced by the smallness of the data set. The units identified as inefficient should increase all outputs in the proportion indicated in the score of efficiency. It was concluded that four of the six inefficient DMUs have R&D expenditures that are too high and therefore should use their resources more efficiently, since these investments are not having the desired reflection in results. Moreover, the Malmquist productivity index allows us to measure the productivity change over the period 2009 to 2011. The results showed an improvement of 34.7% in productivity between 2009 and 2010 and 65.5% between 2010 and 2011. This productivity growth was mainly due to an expansion in the efficiency frontiers, indicating that companies have invested in new technologies in order to improve their productivity.

The authors are conscious of the data limitations and the need for further work in this area. Future work should include the use of other inputs and outputs and DEA extensions to adapt the model to particular circumstances. In order to confirm the importance of incubation in the NTBFs growth, the application of DEA to technology firms that are not incubated, for further comparison, is suggested.

## Figures and Tables

**Table 1:** Data for each DMU for the year 2009.

DMU	Number of employees	Salary costs (€)	R&D investment (€)	Space costs (€)	Product portfolio	Number of clients	Total sales (€)
1	9	215 000	150 000	450	3	3	350 000
2	3	35 000	30 000	220	5	2	45 000
3	7	65 000	30 000	430	3	1	30 000
4	1	15 000	15 000	220	3	1	20 000
5	19	200 000	75 000	1 200	20	150	310 000
6	5	65 000	35 000	350	4	2	100 000
7	5	75 000	50 000	350	1	20	22 000
8	9	175 000	95 000	540	8	95	275 000
9	9	165 000	75 000	380	3	10	215 000
10	3	65 000	22 500	220	5	14	125 000
11	6	125 000	35 000	220	15	12	210 000
12	2	34 500	10 000	220	4	8	60 000
13	10	185 000	75 000	750	10	35	300 000

**Table 2:** Data for each DMU for the year 2010.

DMU	Number of employees	Salary costs (€)	R&D investment (€)	Space costs (€)	Product portfolio	Number of clients	Total sales (€)
1	12	250 000	150 000	740	3	3	400 000
2	3	35 000	30 000	220	6	12	125 000
3	7	65 000	30 000	430	3	3	190 000
4	1	15 000	15 000	220	3	2	25 000
5	20	200 000	75 000	1 200	20	150	350 000
6	9	175 000	55 000	350	5	3	255 000
7	5	65 000	25 000	350	1	35	35 000
8	9	180 000	95 000	540	8	150	325 000
9	9	179 000	75 000	380	3	12	225 000
10	3	65 000	22 500	220	5	13	130 000
11	8	175 000	35 000	475	15	10	225 000
12	3	53 000	10 000	220	4	12	72 000
13	12	220 000	100 000	750	15	40	320 000

**Table 3:** Data for each DMU for the year 2011.

DMU	Number of employees	Salary costs (€)	R&D investment (€)	Space costs (€)	Product portfolio	Number of clients	Total sales (€)
1	13	260 000	15 000	740	3	3	450 000
2	3	35 000	30 000	220	12	15	135 000
3	7	65 000	30 000	430	3	4	120 000
4	1	15 000	15 000	220	3	4	30 000
5	24	250 000	75 000	1 200	20	150	350 000
6	15	265 000	55 000	740	5	4	275 000
7	5	55 000	5 000	350	1	135	85 000
8	11	230 000	95 000	540	12	165	295 000
9	9	175 000	75 000	380	3	11	215 000
10	4	83 000	22 500	220	8	12	132 000
11	15	315 000	55 000	475	15	24	280 000
12	3	54 000	10 000	220	8	17	75 000
13	16	275 000	125 000	750	17	55	385 000

**Table 4:** Correlation coefficients of *inputs* and *outputs*.

	Employees	Salaries	R&D	Space	Sales	Clients	Products
Employees	1,00						
Salaries	0,88	1,00					
R&D	0,65	0,67	1,00				
Space	0,95	0,74	0,54	1,00			
Sales	0,84	0,90	0,61	0,80	1,00		
Clients	0,39	0,20	0,37	0,43	0,21	1,00	
Products	0,61	0,49	0,64	0,51	0,46	0,40	1,00

**Table 5:** Sensitivity analysis with different combinations of inputs and outputs.

	*Inputs *	*Outputs *	Efficiency score mean
Scenario 1	Salaries R&D	Sales, clients, products	0,95
Scenario 2	Salaries R&D	Sales, products	0,90
Scenario 3	Salaries R&D	Sales, clients	0,85
Scenario 4	Salaries R&D	Clients, products	0,73

**Table 6:** Efficiency scores for each DMU in the years 2009, 2010, and 2011.

DMUs	Efficiency scores (ϕ^*^)		
	2009	2010	2011
1	1,00	1,00	1,00
2	1,00	1,00	1,00
3	2,86	1,00	1,47
4	1,00	1,00	1,00
5	1,00	1,00	1,00
6	1,25	1,12	1,56
7	6,25	4,35	1,00
8	1,04	1,00	1,20
9	1,27	1,45	1,54
10	1,00	1,03	1,22
11	1,00	1,00	1,05
12	1,00	1,00	1,00
13	1,00	1,12	1,00

Mean	1,59	1,31	1,16

**Table 7:** DEA-BCC model results.

DMU	Efficiency Score (*ϕ*)	Slacks				Benchmarks
		Salary costs (€)	R&D investment (€)	Product portfolio	Total sales (€)	
1	1,00	0,00	0,00	0,00	0,00	—
2	1,00	0,00	0,00	0,00	0,00	—
3	1,47	0,00	2 000,00	6,38	0,00	1; 2
4	1,00	0,00	0,00	0,00	0,00	—
5	1,00	0,00	0,00	0,00	0,00	—
6	1,56	0,00	2 774,87	0,00	0,00	1; 4; 13
7	1,00	0,00	0,00	0,00	0,00	—
8	1,20	0,00	41 403,27	0,00	0,00	1; 2; 5
9	1,54	0,00	54 333,33	1,78	0,00	1; 2
10	1,22	0,00	0,00	0,00	0,00	1; 10; 5; 12
11	1,05	109 263,91	0,00	0,00	0,00	1; 5; 12
12	1,00	0,00	0,00	0,00	0,00	—
13	1,00	0,00	0,00	0,00	0,00	—

**Table 8:** *Input* and *output* targets for each DMU.

DMU	Salary costs (€)	R&D investment (€)	Product portfolio	Total sales (€)
1	260 000,00	15 000,00	3,00	450 000,00
2	35 000,00	30 000,00	12,00	135 000,00
3	65 000,00	28 000,00	10,79	177 000,00
4	15 000,00	15 000,00	3,00	30 000,00
5	250 000,00	75 000,00	20,00	350 000,00
6	265 000,00	52 225,13	7,78	42 7732,98
7	55 000,00	5 000,00	1,00	85 000,00
8	230 000,00	20 666,67	14,44	354 955,30
9	175 000,00	75 000,00	6,40	331 000,00
10	83 000,00	22 500,00	9,72	160 409,42
11	205 736,07	55 000,00	15,80	295 119,26
12	54 000,00	10 000,00	8,00	75 000,00
13	275 000,00	125 000,00	17,00	385 000,00

**Table 9:** Malmquist index and its components for period 2009-2010.

DMU	2009-2010				
	PTEC	SEC	TEC	TC	*M*_0_
1	1,000	0,600	0,600	1,679	1,008
2	1,036	1,123	1,164	1,438	1,674
3	2,818	1,078	3,037	1,155	3,509
4	1,000	1,000	1,000	1,070	1,070
5	1,000	1,043	1,043	1,009	1,053
6	1,118	0,784	0,876	1,265	1,108
7	1,449	1,012	1,466	1,400	2,053
8	1,041	0,714	0,743	1,534	1,140
9	0,875	0,792	0,692	1,445	1,001
10	0,973	0,998	0,970	1,066	1,034
11	1,000	1,000	1,000	0,931	0,931
12	1,000	1,000	1,000	1,007	1,007
13	0,891	0,843	0,751	1,224	0,919

Mean	1,169	0,922	1,103	1,248	1,347

**Table 10:** Malmquist index and its components for period 2010-2011.

DMU	2010-2011				
	PTEC	SEC	TEC	TC	*M*_0_
1	1,000	1,969	1,969	2,049	4,033
2	1,000	1,000	1,000	1,443	1,443
3	0,678	0,971	0,658	0,996	0,655
4	1,000	0,583	0,583	1,733	1,011
5	1,000	0,676	0,676	1,343	0,908
6	0,719	0,967	0,695	1,298	0,903
7	4,371	0,851	3,719	1,751	6,512
8	0,834	0,946	0,789	1,091	0,861
9	0,939	1,027	0,964	0,999	0,963
10	0,846	0,930	0,786	1,277	1,005
11	0,949	0,532	0,504	1,377	0,695
12	1,000	1,000	1,000	1,573	1,573
13	1,123	0,707	0,793	1,202	0,954

Mean	1,189	0,935	1,088	1,395	1,655
